# A Review of Physiotherapy Techniques Used in the Treatment of Tennis Elbow

**DOI:** 10.7759/cureus.47706

**Published:** 2023-10-26

**Authors:** Anam F Pathan, H V Sharath

**Affiliations:** 1 Pediatric Physiotherapy, Ravi Nair Physiotherapy College, Datta Meghe Institute of Higher Education & Research (DU), Wardha, IND

**Keywords:** lateral epicondylitis, exercises, physiotherapy, elbow pain, tennis elbow

## Abstract

Tennis elbow, a common musculoskeletal disorder also known as lateral epicondylitis, causes pain and tenderness on the outside of the elbow. Although it is frequently linked to repetitive motions, such as those in racquet sports, it can affect people in a variety of occupations and activities. Tennis elbow can be treated conservatively with physiotherapy, which focuses on pain management, functional recovery, and recurrence prevention. The goal of this review is to give a thorough overview of the physiotherapy methods used to treat tennis elbow. In order to determine the most effective treatment options, it is crucial to comprehend the pathophysiology and etiology of tennis elbow from the outset of the study. The assessment and diagnosis of tennis elbow are next covered, emphasizing the importance of physiotherapists in correctly diagnosing the ailment and distinguishing it from other musculoskeletal problems that are comparable to it. This study primarily focuses on the numerous physiotherapy therapies for tennis elbow, which may include but are not limited to, and the section examines the use of manual treatments to treat pain and enhance joint function, including joint mobilizations, soft tissue massage, and myofascial release. Exercise rehabilitation covers the value of tailored workouts to bolster the weak muscles and enhance the elbow joint's biomechanics. Numerous workout regimens are covered, such as eccentric training and progressive resistance exercises, as well as modalities. Therapeutic agents frequently make use of modalities such as ultrasound, laser therapy, and cryotherapy. It may also make use of complementary therapeutic agents such as taping and bracing. In summary, this in-depth analysis highlights the crucial role that physical therapy plays in the treatment of tennis elbow. It seeks to give practitioners a useful tool for enhancing the care and results of patients with this common and crippling ailment by summarizing the most recent research and best practices in physiotherapy approaches.

## Introduction and background

The extensor tendons in the dorsal forearm, which attach at the lateral epicondyle of the humerus, experience a painful condition known as tennis elbow. Nearly 90% of the time, the extensor carpi radialis brevis (ECRB) muscle is the afflicted tendon. With an annual prevalence of 10 to 30 cases per 1,000 adults and a peak incidence between the ages of 35 and 55, it is a frequent cause of elbow pain. The majority of the time, a clinical diagnosis of lateral epicondylitis (LE) may be obtained. However, when the diagnosis is not quite obvious, additional research may be needed. Anyone who engages in repetitive gripping and wrist extension activities, including tennis players as well as people working in manual labor, using a computer to type, or even engaging in hobbies like gardening or painting, is susceptible to developing this illness [1].

Tennis elbow is characterized by chronic discomfort and tenderness on the outside of the elbow, which makes seemingly simple activities like raising a coffee cup or shaking hands unbearably uncomfortable. Beyond only being uncomfortable, tennis elbow can have long-lasting effects that interfere with a person's ability to function at work and in daily life. The fact that the disease is prevalent during a stage of life when people are actively seeking employment has made it a significant contributor to absenteeism (most often extended). Tennis elbow therefore has a significant financial impact [2]. 

Pathophysiology

The pathophysiology is primarily caused by chronic overuse or repetitive strain, which results in microtears or degeneration of the extensor tendon attachment at the lateral epicondyle. Tennis elbow is complicated, involving biomechanical, occupational, and individual aspects, which makes diagnosis and treatment challenging. Tennis elbow is a frequent and painful condition that affects the outside of the elbow and is medically referred to as lateral epicondylitis [3]. Tennis elbow, despite its name, is a condition that can affect anyone who uses their forearm muscles and tendons regularly, particularly in tasks that require gripping and twisting actions. Pain and discomfort on the outside of the elbow, which may extend down the forearm, are the usual symptoms of this illness. It happens when overuse or strain leads to inflammation or injury of the tendons that connect to the lateral epicondyle, the bony bump on the outside of the elbow. Tennis elbow might develop as a result of pursuits including tennis, gardening, woodwork, or even high computer mouse use [4].

The outer region of the elbow is affected by the lower extremity as well as the "tennis elbow," which is an excruciating condition. It is a form of tendinopathy, meaning the tendons connecting to the LE are bony projections. The specific area referred to is situated on the outer part of the elbow and is damaged and degenerated. Due to repetitive motions, the ECRB muscle is this syndrome's most frequently affected muscle. Pain and tenderness experienced on the outer part of the elbow, diminished grip power, and trouble completing tasks requiring wrist and hand movements are all signs of this illness [5]. The lateral elbow has bony and ligamentous structures supporting the joint and five muscles serving as the origin of the dorsal forearm's musculotendinous attachments [3]. Tennis elbow was shown to occur between 1% and 2% of the time in epidemiological studies. Stage 1 of LE contrasts with stage 2 of angiofibroblastic hyperplasia, identified by high cell numbers, blood vessel hyperplasia, and collagen fiber disintegration. Therefore, tendinosis may be a better description than tendinitis. Stage 3 lesions may involve partial- or full-thickness tendon rips, while stage 4 may include fibrosis and calcification [6].

The most typical age range for beginning lateral epicondylitis is between the third and fourth decades of life. Women and those who report nerve complaints are more likely to have a poorer short-term prognosis in lateral epicondylitis P.T. therapy. This condition is distinguished by chronic aches on the elbow's outer side brought on by frequent usage. The elbow should be examined using the normal "look, feel, move" method and the provocative tests of Mill and Maudsley to validate any clinical suspicion [7,8]. Rest and physical therapy exercises emphasizing strengthening should be the primary lines of treatment. For LE, numerous therapies are available, ranging from medical interventions such as surgery and medication to physical therapy, including modalities, exercise, and manual therapy. Because of the lack of side effects, splinting is advised in the disorder's early stages. The examiner performs Mill's test for LE by gently pronating the forearm, flexing the wrist, and extending the elbow while using your thumb to palpate the patient's lateral epicondyle. A positive test will reproduce the pain around the lateral epicondyle. The third finger of the patient's hand is extended against the examiner's resistance during the Maudsley test for lateral epicondylitis, which is performed with the patient's forearm pronated and elbow flexed at 90 degrees. If the test is positive, the pain near the lateral epicondyle will return [9,10].

## Review

Study selection

**Figure 1:**
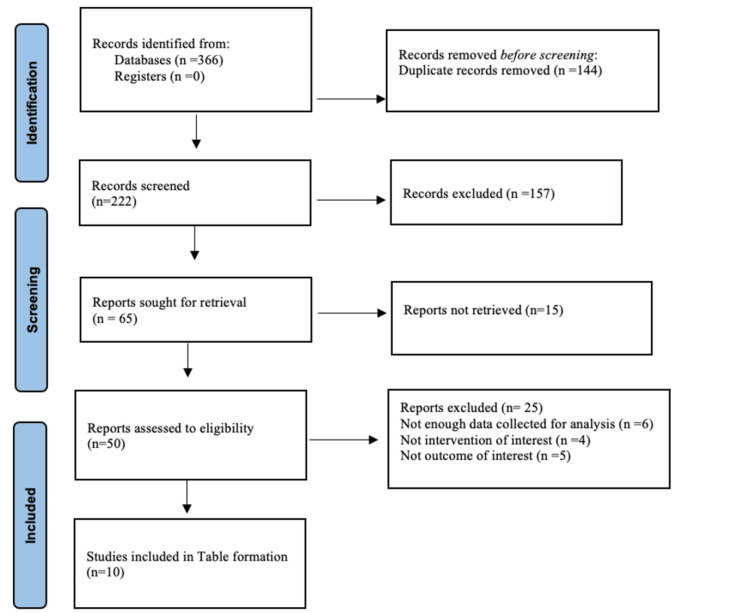
The Preferred Reporting Items for Systematic Reviews and Meta-Analyses (PRISMA) diagram showcasing the study selection process

English-language literature was searched on Google Scholar and PubMed for randomized and non-randomized clinical trials to evaluate the impact of various physiotherapeutic interventions on lateral epicondyle in patients with lateral epicondylitis between 2004 and 2021. For this review, we included original articles, systematic reviews, meta-analyses, and randomized controlled trials. Keywords for this study were "Cyriax", "myofascial release", "tendinitis", "trigger points", "tennis elbow", and "degeneration", and in the same search, Boolean terms used were "WITH", "AND" and "OR". The flow diagram of the review is given below in Figure 1.

Physiotherapy interventions

Manual Therapy and the Cyriax Method

Deep transverse frictional (DTF) massage, which was initially endorsed by Cyriax, has been the subject of very few investigations. In a four-week treatment period, 12 sessions of transverse frictions were compared to a corticosteroid injection in Ahmed et al.'s randomized controlled experiment. Subjective and objective markers improved in the steroid group after six weeks, but there were no differences between the groups at the 12-month follow-up. The authors concluded that friction massage was no more effective at treating tennis elbow than an injection [11-12].

The Cyriax technique and deep friction massage (DFM), sometimes called cross friction massage, is a type of connective tissue massage created by James Cyriax. Deep friction massage aims to keep the soft tissue structures of ligaments, tendons, and muscles mobile and to keep adherent scars from developing. Cyriax treats LE using DTF and Mill's manipulation, whichever is executed. In the Cyriax technique, elements must be integrated and worn in an appropriate sequence. Patients must attend the program three times per week for four weeks. Deep transverse friction is given with the tip of the thumb. Pressure was exerted from behind the teno-osseous junction [13-15]. It aims to stop aberrant fibrous adhesions and scarring.

Mill's Manipulation

Mill's manipulation consists of a low-amplitude, high-velocity elbow extension push after the entire range of elbow extension has been exhausted, a passive movement carried out at the maximum capacity of elbow extension. Using 0.4% dexamethasone iontophoresis and Cyriax-type exercises greatly improved LE [16,17]. Treatment with corticosteroid injections was more beneficial than Cyriax physiotherapy. Cyriax friction massage is a well-known and commonly utilized therapy in chronic pain management [18-19].

Myofascial Release Technique

Applying a regulated force to soft tissue myofascial structures in a specified direction to stretch the tissues to restore or enhance normal mobility in a restricted location [20]. Myofascial release restores optimum length, lowers discomfort, and improves function by providing a low-load, prolonged stretch to the complex [21,22]. Tender spots, myofascial trigger points (MTrPs), and pain brought on by utilizing the arms, particularly when grabbing and carrying large objects, are typical clinical symptoms.

The muscles causing myofascial pain typically have one or more MTrPs. The most painful area of skeletal muscle, an MTrP, is likely caused by an accumulation of hypersensitive nociceptors. A special questionnaire for measuring the health state of patients with LE is provided [23-25]. The questionnaire is divided into two subscales: pain, which has five questions, and function, which has 10 items. The scores range from 0 (the best score) to 100 (the poorest score). Self-myofascial release (SMFR) offers potentially beneficial impacts for both athletes and the general public, including improved malleability and recuperation. Myofascial release therapy lacks sufficient data to justify its use for persistent musculoskeletal pain. Myofascial unwinding (MFU), also known as the indirect myofascial release technique, is defined as "a manual technique incorporating constant feedback to the osteopathic practitioner that includes passively moving a portion of the patient's frame in response to the experience of movement [26-28].

Laser Treatment

In the UK, physiotherapists seldom utilize this to treat tennis elbow. The inconsistent findings from earlier studies show that this therapy modality's effectiveness in the short term is debatable. When compared to a placebo, there is currently no proof that laser therapy has any long-term effects [29].

Short-Wave Diathermy With Pulses

Despite there being no definitive evidence about its usefulness in the care of tennis elbow, this method was utilized by less than 10% of physiotherapists in Greenfield & Webster's study [30].

Ultrasound

Just under 50% of physiotherapists treating tennis elbow utilize pulsed and continuous ultrasound [6]. However, there is disagreement on the treatment's general efficacy for musculoskeletal problems. Using a variety of outcome measures, trials comparing pulsed ultrasound with a placebo are observed to have a variety of effects. There were no significant changes in results between groups when compared to alternative modalities, such as injections or transcutaneous electrical nerve stimulation (TENS), and there is scant data to support its efficacy. Only one study, which employed ultrasound in conjunction with a steroid coupling gel, explored the utility of phonophoresis. There were no more advantages to utilizing a steroid coupling gel than to using ultrasound alone after nine therapy sessions [31].

Laser Therapy

In individuals with lateral epicondylitis who have persistent symptoms, high-intensity laser therapy is a useful therapeutic approach for reducing pain and enhancing the quality of life (36-item short-form health survey physical component). However, the 36-item short-form health survey that measured grip strength, hand function, and quality of life did not reveal any appreciable differences between high-intensity laser therapy and other treatments.

A summary of the articles reviewed for physiotherapy treatment in lateral epicondylitis is given in Table 1.

**Table 1:** A summary of the articles reviewed for physiotherapy treatment in lateral epicondylitis Le: lateral epicondylitis; MFR: myofascial release; LLLT: low-level laser therapy; MTrPs: myofascial trigger points; RMS: repetitive motion strain

Sr.No	Author, year	Study design	Journal	Main finding
1.	Ahmad et al.,2013 [5]	Review article	Bone Joint J	LE, also known as "tennis elbow," is a common illness that typically affects patients between the ages of 35 and 55. It is usually self-limiting, but in some cases, it can cause persistent symptoms that are resistant to therapy. The mechanism of disease, symptoms and indicators, investigations, current management methods, and prospective new therapeutics are all included in this study.
2.	Duncan et al., 2019 [7]	Review article	Br J Hosp Med (Lond)	LE is a frequent condition that affects both men and women, affecting between 1% and 3% of individuals. It compiles the most recent research on LE to assist clinicians in performing assessments and making treatment decisions. The recommendations provided are in line with current clinical practice.
3.	Stasinopoulos et al., 2004 [13]	Review article	Br J Sports Med	Tennis elbow, also known as LE, is a very frequent arm lesion with a well-defined clinical presentation that has a considerable impact on the population. To control this illness, numerous therapy options have been recommended. Cyriax physiotherapy is one example. This intervention's effectiveness and stated effects are examined.
4.	Viswas et al., 2012 [14]	Randomized clinical trial	ScientificWorldJournal	Both the supervised exercise program and Cyriax physiotherapy were found to significantly reduce pain and improve function. Supervised exercise was more effective than Cyriax physiotherapy.
5.	Ajimsha et al., 2012 [20]	Randomized, controlled, single-blinded trial	Arch Phys Med Rehabil	During weeks four and 12, the MFR group outperformed the control group. (P.005), patients in the MFR group reported a significant reduction in pain and functional impairment compared to the control group. This improvement persisted at week 12. MFR is a better way to relieve lower extremity pain for professionals working on computers.
6.	Chang et al., 2010 [22]	Review article	Photomed Laser Surg	We believe that utilizing LLLT interminably LE MTrPs or tender points may be a concern and significantly increase results.
7.	Lin et al., 2012 [23]	Case study	Evid Based Complement Alternat Med	Percutaneous soft tissue release may be utilized to prevent the recurrence of chronic reiterative LE. Provided treatment options included medication, physical therapy, or a steroid injection, are ineffective.
8.	Nagrale et al., 2009 [29]	Randomized clinical trial	J Man Manip Ther	The focus of this research is to show that it has been reported that Cyriax physiotherapy is more effective than phonophoresis and exercise when it comes to the treatment of LE.
9.	Meltzer et al. 2010 [30]	Case study	J Bodyw Mov Ther	Following RMS, MFR treatment normalized apoptotic rate and cell shape, both of which were comparable to the alterations found. These in vitro investigations add to the cellular evidence foundation required to adequately describe the clinical efficacy of manual manipulative therapy.
10.	Ahmed et al., 2021 [31]	Comparative study on clinical trials	J Pak Med Assoc	Patients suffering from lateral epicondylitis can greatly benefit from the use of the Mulligan and Cyriax techniques. These methods were found to effectively enhance performance and alleviate discomfort, leading to a better quality of life for those afflicted with this condition.

Discussion

Tennis elbow, a common overuse ailment affecting the tendons in the elbow, is managed comprehensively with the help of physiotherapy. Different methods have been used to treat the symptoms and encourage healing. Eccentric exercise treatment, which involves carefully extending the injured muscle to increase its strength and flexibility, is one of the most often employed techniques. By encouraging tissue remodeling and accelerating the healing process, this approach has demonstrated promising benefits for lowering pain and enhancing function. Also effective at improving joint mobility, easing muscle tension, and reducing pain are manual therapy techniques like soft tissue mobilization and joint mobilization. These methods are frequently used in thorough physiotherapy programs that are individualized for each patient's needs and emphasize a holistic approach [30].

Treatment for tennis elbow involves a lot of witchcraft and pseudoscience. With a natural history of between 10 and 18 months, it is common for lateral epicondylitis to resolve spontaneously. In the vast majority of individuals, the illness will eventually improve, and symptoms are typically well-controlled by non-operative treatments like activity modification and physiotherapy. Tennis elbow is a widely recognized term utilized to describe tendinosis, a situation that impacts the tendons in the elbow that almost frequently affects the ECRB tendon, which has both a degenerative and a compromised healing process. The pronator teres, flexor carpi radialis, medial elbow joint, and posterior triceps are the specific elbow abnormality sites. Energizing the unhealthy tendinosis tissue that causes pain is the aim of nonoperative treatment [31].

The strains (eccentric) that a tendon can endure can be maximized by optimizing tendon training and recuperation. It may be concluded that mobilization with motion (MWM) is a promising intervention method for the treatment of patients with lateral epicondylalgia. In individuals with lateral epicondylalgia, pain-free grip strength is a more sensitive outcome measure than maximum grip strength. Myofascial methods, by loosening constricted fascia, may alter the duration and intensity of vasospastic episodes encountered in primary Raynaud's phenomenon [32].

According to a recent study, the efficacy of physical therapy techniques was examined, beginning with a summary of the overall efficacy of the physiotherapy approaches examined. The methods have been effective in reducing discomfort, enhancing function, and assisting in the rehabilitation of people with tennis elbow. Use particular figures and studies to back up your claims [33]. Personalized treatment programs also have the significance of tailored treatment strategies, which should be emphasized. Tennis elbow is not a problem that can be treated in a uniform manner; thus, physiotherapy effectiveness largely rests on adjusting interventions to the particular requirements and traits of each patient. Physiotherapists need to talk about how the choice of techniques is influenced by variables such as the severity of the ailment, patient age, activity level, and comorbidities and give particular attention to eccentric exercises because they have become a pillar of tennis elbow rehabilitation [34].

The diverse nature of treatment interventions is highlighted by a physiotherapy technique used in the management of tennis elbow. The main element is exercise therapy, which emphasizes eccentric movements and thorough rehabilitation plans meant to improve muscle strength and flexibility. Utilizing specialized exercise routines promotes tissue repair and improves overall elbow health while improving functional outcomes and pain management. The use of targeted massage and soft tissue mobilization, two manual treatment techniques that have the potential to reduce muscular tension, promote blood flow, and speed up the healing process, are also essential elements. However, there is still more research needed to determine the long-term effectiveness of electrotherapy modalities, including ultrasound and shockwave therapy, for the treatment of tennis elbow patients' pain and tissue healing. To further emphasize the importance of resolving underlying movement dysfunctions and advocating ergonomic habits to prevent re-injury and guarantee long-term rehabilitation success, biomechanical tests, and patient education tactics have been integrated into treatment plans [35-36].

In addition, the use of therapeutic techniques like extracorporeal shockwave therapy, laser therapy, and ultrasound has become more popular in the management of tennis elbow. These techniques are used to reduce discomfort, speed up tissue recovery, and enhance the affected area's general functionality. Low-level laser therapy, on the other hand, has demonstrated analgesic and anti-inflammatory effects. For instance, ultrasound therapy has been found to stimulate tissue repair and reduce inflammation by increasing local blood flow. A non-invasive treatment approach that can speed up the healing process and encourage tissue regeneration is extracorporeal shockwave therapy. In order to facilitate the patient's recovery, physiotherapists carefully choose and incorporate these techniques into thorough treatment programs, taking into account the severity of the ailment and the individual's unique demands.

Limitations

Despite the review's depth on physiotherapy methods for tennis elbow, some limitations must be addressed. First off, the generalizability of the results may be impacted by the variation in the severity and chronicity of tennis elbow among patients. Depending on a patient's age, degree of activity, and general health, different physiotherapy approaches may be more or less helpful. Furthermore, it may be difficult to come to firm conclusions on the best way to treat tennis elbow due to the variety of methodologies and procedures used throughout the many studies included in the study. Additionally, the dearth of long-term follow-up information in many studies may make it more difficult to determine how long-lasting and sustainable the observed treatment benefits will be.

The review's accuracy is further limited by its dependence on subjective outcome indicators like self-reported pain and functional evaluations. Some studies may contain inherent biases due to the lack of standardized, objective metrics, which jeopardizes the validity of the results. Additionally, the possibility of publication bias, which occurs when research with significant findings is more likely to be published, can influence how the effectiveness of particular physiotherapy approaches is ultimately interpreted. This bias may cause an overestimation of the advantages of particular interventions, restricting our ability to fully comprehend the effectiveness of different physiotherapy methods in the treatment of tennis elbow. Future research efforts should strive to adopt a more standard methodology, include long-term follow-up assessments, and use impartial methods in light of these limitations.

## Conclusions

The literature review highlights the importance of physiotherapy therapies in the treatment of tennis elbow. Numerous methods, including manual therapy, the Cyriax method, deep friction massage, Mill's manipulation, myofascial release technique, laser treatment, short-wave diathermy with pulses, and ultrasound, have shown promise in reducing pain, enhancing function, and accelerating the repair of damaged tissue. The limitations noted, such as the variation in patient characteristics, the heterogeneity of methodology, and the dependence on arbitrary outcome measures, however, underscore the need for additional study to develop more uniform procedures and unbiased evaluations. It is crucial to have a thorough grasp of the long-term effectiveness, specific treatment strategies, and effects of physiotherapy therapies on patients' overall functional capacity. The area of physiotherapy can improve its contributions to the treatment and rehabilitation of individuals with tennis elbow by addressing these constraints and promoting a more rigorous approach to research.
